# Pros and cons of using rapid sequence induction in all cases requiring general anesthesia

**DOI:** 10.1186/s40981-024-00720-5

**Published:** 2024-06-06

**Authors:** Keisuke Yoshida, Atsushi Takizuka, Ko Kakinouchi, Satoki Inoue

**Affiliations:** 1 Department of Anesthesiology, Fukushima Medical University School of Medicine, 1 Hikariga-Oka, Fukushima, 960-1295 Japan; 2 Department of Anesthesiology, Yamashita Hospital, Ichinomiya, Aichi Japan; 3 Department of Anesthesiology, Hiratsuka City Hospital, Hiratsuka, Kanagawa Japan

To the Editor,

Rapid sequence induction (RSI) is a technique of general anesthesia induction in which anesthetics (sedatives, muscle relaxants, and/or analgesics) are simultaneously administered intravenously; then, after approximately 60–90 s without manual ventilation, tracheal intubation is performed. The main aim of RSI is to minimize the risk of gastric content aspiration; thus, it performed in patients with a high risk of aspiration, especially those with full-stomachs [1, 2]. However, whether RSI should be the first choice in all cases requiring general anesthesia has yet to be elucidated.

One of the advantages of RSI is that it has a low risk of gastric insufflation and vomiting, which was its original purpose. In addition, since RSI does not require volatile anesthetics, there is no concern about environmental contamination from volatile anesthetics. Another advantage of RSI is that the absence of ventilation leads to reduction in aerosol generation; thus, RSI is recommended for tracheal intubation in patients with COVID-19 [3]. These advantages support the use of RSI in all cases requiring general anesthesia in our current post-pandemic society.

On the other hand, there are several disadvantages to RSI. The first is a delay in airway intervention due to the absence of ventilation (approximately 60–90 s [2]). This can result in a life-threatening delay in cases of unanticipated difficult airway (DA). Another disadvantage of RSI is that the necessary doses of anesthetics tend to be high, which may increase the risk of hemodynamic changes. In addition, many airway management guidelines state that restoration of consciousness and spontaneous breathing are the leading options when unanticipated DA occurs after administration of anesthetics [4]. However, the administration of high doses of anesthetics is also associated with prolonged awakening and need for high dose antagonists (e.g., sugammadex) in emergency situations. Thus, RSI is not suitable for scenarios where DA or hemodynamic changes are anticipated.

Given the benefits of RSI, which reliably protects healthcare providers against aerosol exposure, and its drawback of potential life-threatening delays in extremely rare unforeseen DA cases, one could argue for the use of RSI as the primary method for general anesthesia induction in routine cases. Although a slight deviation from the definition of RSI, which includes no ventilation, a single, very small, ventilation (even smaller than so-called modified RSI [5]) immediately after falling asleep to ensure that ventilation is possible may be an optimal option, maximizing the benefits of RSI while addressing potentially life-threatening delays. Of note, it is essential to weigh the benefits against the drawbacks when deciding whether to use RSI as the primary method to induce general anesthesia. However, in our current post-pandemic society, a paradigm shift in general anesthesia induction may be imminent as the times change, which is represented by widespread use of video laryngoscopes, sugammadex, and advanced supraglottic airway devices. This topic presents various pros and cons worth considering.

## Data Availability

Not applicable.
